# Clinician voices on ethics of LLM integration in healthcare: a thematic analysis of ethical concerns and implications

**DOI:** 10.1186/s12911-024-02656-3

**Published:** 2024-09-09

**Authors:** Tala Mirzaei, Leila Amini, Pouyan Esmaeilzadeh

**Affiliations:** Information Systems & Business Analytics, College of Business, Florida International University, 11200 S.W. 8th St., Room RB 250, Miami, FL 33199 USA

**Keywords:** Artificial Intelligence, LLM, Ethics, Theme, Thematic analysis

## Abstract

**Objectives:**

This study aimed to explain and categorize key ethical concerns about integrating large language models (LLMs) in healthcare, drawing particularly from the perspectives of clinicians in online discussions.

**Materials and methods:**

We analyzed 3049 posts and comments extracted from a self-identified clinician subreddit using unsupervised machine learning via Latent Dirichlet Allocation and a structured qualitative analysis methodology.

**Results:**

Analysis uncovered 14 salient themes of ethical implications, which we further consolidated into 4 overarching domains reflecting ethical issues around various clinical applications of LLM in healthcare, LLM coding, algorithm, and data governance, LLM’s role in health equity and the distribution of public health services, and the relationship between users (human) and LLM systems (machine).

**Discussion:**

Mapping themes to ethical frameworks in literature illustrated multifaceted issues covering transparent LLM decisions, fairness, privacy, access disparities, user experiences, and reliability.

**Conclusion:**

This study emphasizes the need for ongoing ethical review from stakeholders to ensure responsible innovation and advocates for tailored governance to enhance LLM use in healthcare, aiming to improve clinical outcomes ethically and effectively.

**Supplementary Information:**

The online version contains supplementary material available at 10.1186/s12911-024-02656-3.

## Introduction

The development of Large Language Models (LLMs) marks a significant advancement in integrating deep learning techniques within natural language processing (NLP), enhancing the field’s capabilities far beyond traditional methods. This represents a deepening of the synergy between cutting-edge artificial intelligence (AI) technologies and foundational NLP approaches.These generative AI models, trained on vast datasets, have demonstrated remarkable proficiency in generating text virtually indistinguishable from human-authored content [1]. This transformative potential extends across diverse domains, including healthcare, where they offer the capacity to process, analyze, and generate insights from extensive textual healthcare data. Notably, recent studies have shown that technologies like ChatGPT (an LLM developed by OpenAI) outperform physician-patient communication in terms of both quality and empathy [2].

However, integrating LLMs into sensitive areas like healthcare has several challenges [3]. These models may occasionally produce inaccurate or biased responses [4]. In medical informatics, such inaccuracies can have far-reaching consequences, potentially resulting in physical and psychological harm, as well as inappropriate changes in treatment or patient adherence [5, 6]. It is essential to recognize that using LLM-driven recommendations in healthcare differs significantly from other sectors due to the highly sensitive nature of health information and the heightened vulnerability of consumers to potential medical errors [3]. LLMs, designed for plausibility rather than absolute accuracy, do not inherently verify the truthfulness of their output [7, 8]. Additionally, while valuable, their tuning through human feedback is not infallible [9, 10].

The primary objective of this research is to investigate and elucidate the ethical complexities inherent in integrating LLMs within healthcare settings, with a specific focus on the perspectives of clinical practitioners. Our objective is to categorize and critically analyze the ethical challenges that emerge from the deployment of LLMs in healthcare, thereby contributing to a more informed understanding within the field. Overlooking these ethical concerns could result in missed opportunities to harness LLMs effectively, including optimizing processes like triage, screening, and treatment administration [11–13], while also offering the potential to democratize healthcare access through the direct provision of AI-driven healthcare services to patients’ homes. [14–16] Ethical lapses or misinterpretations may result in societal resistance or the formation of skewed regulations and policies, thereby hindering the advancement and acceptance of vital data science applications in healthcare [8]. This study underscores the transformative potential of LLMs in healthcare, contingent upon their implementation being guided by a thorough understanding of the ethical implications.

## Material and method

This study aimed to elucidate emergent themes within the “medicine” subreddit (https://www.reddit.com/r/medicine/), a digital platform specifically for medical professionals. The subreddit comprises more than 465,000 members, including self-identified physicians and other healthcare professionals from across the globe. This subreddit mandates that all users must set a flair accurately reflecting their role in the healthcare system. All posts and comments are in the English language. We considered this subreddit more suitable for academic research than other social media platforms, such as Twitter/X, due to its community structure and the nature of interactions within the platform. The specialized community in this subreddit enables us to easily find and engage with content that is highly relevant to the topic of the study. The format of Reddit supports longer, more in-depth discussions. In addition, this subreddit has a dedicated moderator and established rules for posting, so off-topic posts and spam are often quickly removed.

**Fig. 1:**
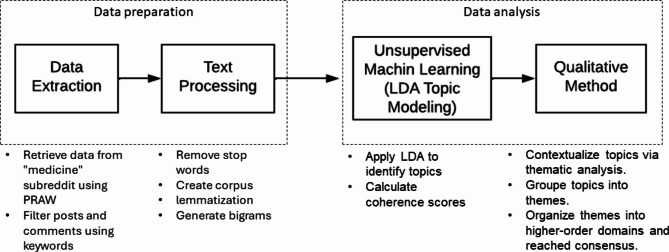
Method overview

We adopted the Sequential Explanatory Method [17], which represents a mixed methods research design characterized by an initial phase of quantitative data collection and analysis, followed by a subsequent phase of qualitative analysis. The research design pipeline is shown in Fig. 1.

We initially applied an unsupervised machine learning technique to uncover prominent latent topics within the large-scale unstructured data. Subsequently, we employed thematic analysis to further refine these topics. This involved a detailed examination of the data, including the inductive development of themes based on the latent topics identified by the machine learning algorithms [18, 19]. Our primary focus was on individual posts, each typically hosting multiple user comments pertaining to the thread’s subject matter (e.g., “ChatGPT in medicine” posted on February 21, 2023).

### Data collection

Data for this study were collected over 12 months, from November 2022 to November 2023. This timeframe corresponds with the release of ChatGPT by OpenAI in November 2022, a refined version of the GPT-3 LLM optimized for conversational responses. The period is characterized by rapid user adoption of LLMs, providing a relevant context for examining its impact and usage [20]. We employed a selection of keywords to specifically extract the viewpoints of the subreddit members regarding ethical issues of integration of LLM and AI in healthcare. To select the keywords, we included broad terms such as “artificial intelligence”, “Large Language Model” and “ethical AI”, ensuring we cover variations and abbreviations of these terms to consider different ways of expressing the same idea. We included the specific model names such as “ChatGPT” and “GPT-3”. We also considered related concepts such as “privacy” and “transparency” to gather the relevant information about the topic. The complete list of keywords is provided in the supplemental material. For data extraction, we utilized the Python Reddit API Wrapper (PRAW) to interface with Reddit’s API, capturing post URLs, timestamps, the textual content of the posts, and the text from associated threads.

### Data processing techniques

We employed the Natural Language Toolkit (NLTK) library (version 3.8.1) for processing textual data [21]. This process involved eliminating stop words, breaking down paragraphs into sentences, and further decomposing sentences into individual words or tokens as well as lemmatization to reduce words to their base form [18, 22]. Additionally, our preprocessing included generating sequential word combinations, namely bigrams (e.g., mental disorder), as part of our feature set to capture more nuanced linguistic structures.

### Unsupervised machine learning

We utilized the Latent Dirichlet Allocation (LDA) [23] approach, a well-established technique in NLP, social media analytics, and information retrieval [19]. LDA, an unsupervised probabilistic model, identifies topics by detecting underlying semantic patterns in a substantial text corpus [24]. Based on the data itself, the algorithm produces frequently mentioned pairs of words, the pairs of words that co-occur, and latent topics and their distributions over topics in the document. We calculated coherence scores to assess the validity of our topic model and identify the optimum number of topics. These scores measured the semantic similarity among words within a topic, indicating our model’s interpretability and thematic consistency. This similarity was determined by representing words as vectors based on their co-occurrence relationships. The coherence score was then calculated as the arithmetic mean of these similarities [25]. High coherence scores suggested meaningful thematic groupings, while lower scores may point to topics formed by statistical inference rather than actual thematic coherence. We employed Gensim (version 4.3.2; RARE Technologies Ltd) [26], an open-source Python library dedicated to topic modeling [19], for practical implementation.

### Qualitative analysis

We employed a qualitative thematic analysis to complement and contextualize the LDA model findings. Our interpretative analysis adhered to a thematic analysis model [27]. The research team comprised three subject matter experts in the field of health informatics. Each researcher conducted a thorough review of a selection of at least five posts and their corresponding comments to identify and familiarize themselves with the emerging topics. A priori themes were utilized as the initial coding template based on the Governance Model for AI in Healthcare (GMAIH) [14]. Additional themes were generated for topics that did not readily adhere to a priori themes. Working independently, we assigned thematic names to the topics, ensuring they accurately represented the post content. We then critically assessed the initial codes for their alignment with the identified topics. In this assessment, we compared the theme names that each of us individually assigned to the labeled topics. This process continued until a unanimous consensus was achieved among all three researchers.

## Results

**Fig. 2:**
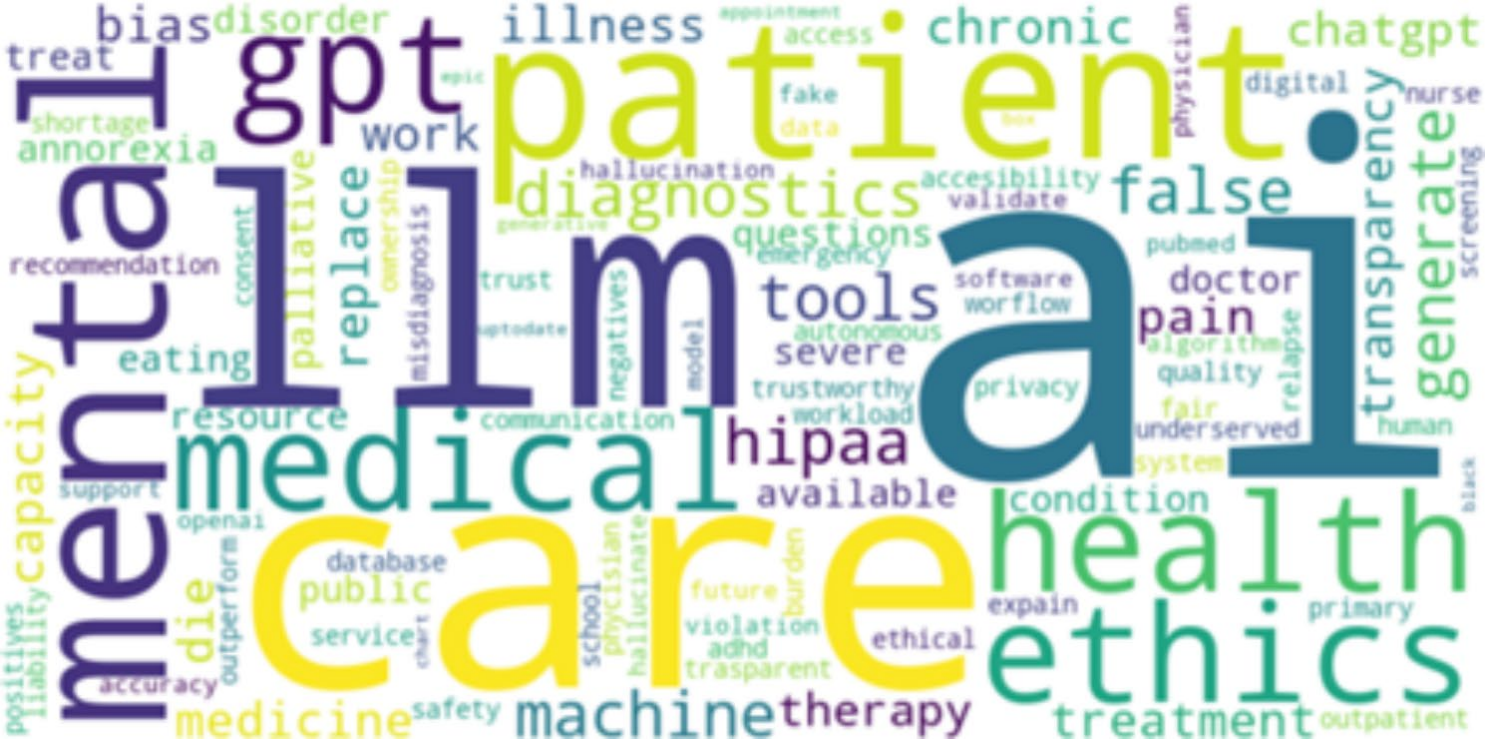
The word cloud of the most popular unigrams

After processing all raw data, our final dataset included 3049 relevant posts and comments. We identified the most popular unigrams and bigrams regardless of the grammar structure of the words. Figure 2 shows a visualization of the most popular unigrams.

**Fig. 3:**
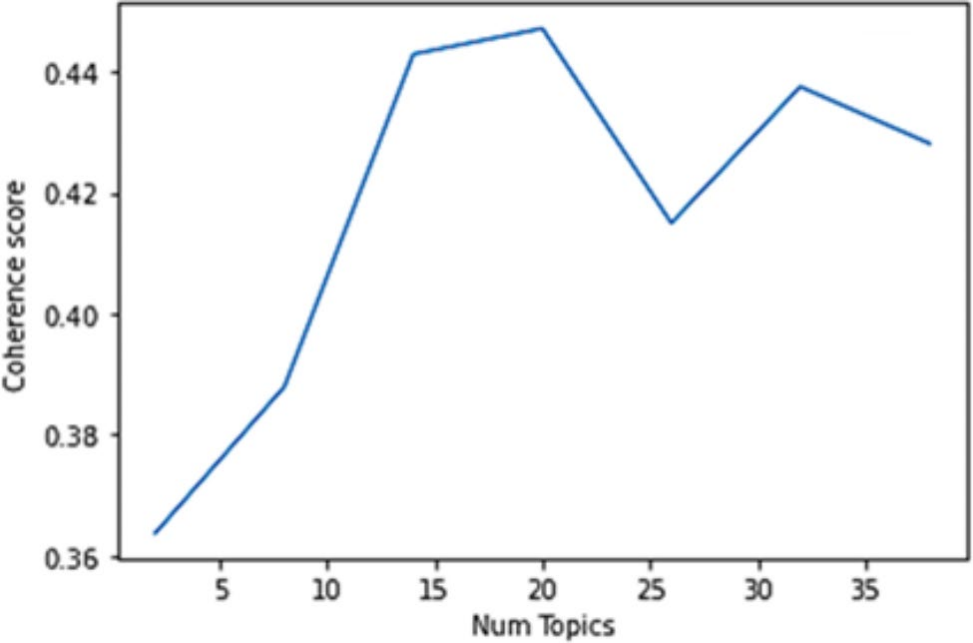
Highest coherence score achieved for the model with 20 topics

In alignment with prior studies [25, 28, 29], we calculated the C_V_ coherence score [25, 30] to ascertain the optimal number of topics tailored to our dataset. Through this analytical process, the LDA model suggested that a configuration of 20 topics would yield a high coherence score for our data. We present the variation in coherence scores as a function of the total number of topics in Fig. 3. Our analysis demonstrates that coherence scores range from 0.36 to 0.45, with the highest coherence for the model that includes 20 topics.

Next, three researchers completed the thematic analysis on the initial 20 topics generated as the results of the LDA analysis. This initial comparison resulted in an inter-annotator agreement rate, calculated using Fleiss’s Kappa, which equaled 0.66. Discrepancies primarily arose from interpretational nuances, exemplified by one researcher favoring a more abstract label (e.g., “Trustworthiness of AI”) while another leaned towards a more specific label (e.g., “Trust in use of AI for health-related information-seeking”). Upon closer examination, we observed conceptual similarities among several topics, leading us to converge them into broader themes with the agreement from all three researchers to ensure the topics corresponded meaningfully under one theme. An iterative process of comparison and consensus-building was employed to resolve discrepancies and achieve a unanimous agreement on thematic categorization. This method is in accordance with previous studies [25, 28, 29] that highlight the necessity of human intervention to clarify themes and reduce overlaps beyond what statistical measures alone can achieve. This process resulted in consolidating the initial topics into 14 distinct themes, as detailed in Table 1. For example, three separate topics about LLM role in “communications about home visit and insurance”, “patient_provider interactions” and “communications about Narcan and relapse case” were merged into one broader theme as “LLM-enhanced healthcare communications”. An extended version of Table 1 is provided in the supplemental material that provides more details about the topics, extended definition of themes, potential ethical concerns, and direct quotes from the subreddit posts. This approach provides a framework for situating our findings within the ongoing discourse on LLM ethics in healthcare.

**Table 1:** Themes and subthemes extracted from the data

Theme	Description	Examples of quotes
1. LLM-Enhanced Healthcare Communication	Explores ethical concerns surrounding the use of LLM in facilitating communication for healthcare purposes, including patient-provider interactions and discussions of preventive measures.	“-“The use of AI [LLMs] to enhance doctor-patient communication could improve outcomes, but we must consider how to prevent harm due to potential miscommunication or misunderstanding.”
2. LLM in Nursing and Care Quality Improvement	Focuses on the ethical aspects of LLM’s role in nursing, such as improving job performance and care quality, while considering the reduced decision-making burden on care providers.	-“I had a nursing Educator during my RN residency program describe some technology similar to this [LLMs] and that it would eventually be able to chart my shift assessment for me.”
3. Ethical Monitoring of LLM Coding in Healthcare	Discusses the need for ethical guidelines and standards in developing and coding LLM tools in healthcare.	-“For LLM coding in healthcare, robust oversight must warrant algorithms are free from biases and respect patient diversity… I fear AI optimized for efficiency could worsen issues if deployed in medical coding without a framework ensuring decisions are fair.”
4. Privacy Ethics in LLM-Enabled Medical Data	Highlights the ethical concerns related to privacy and data security in the context of LLM accessing sensitive medical lab results.	-“Effective anonymization measures are essential for using private health data to train diagnosis LLMs that protect patient confidentiality.”
5. LLM in Emergency Care: Ethical Perspectives	Examines the ethical considerations in using LLM for emergency and outpatient treatment, emphasizing patient safety and treatment efficacy.	-“These [LLM] tools might aid emergency triage, but safeguards are needed to prevent over-reliance on imperfect algorithmic assessments when immediate care is critical.”
6. Ethical Challenges in LLM-Powered Rural Healthcare	Explores ethical questions about using LLM to enhance healthcare accessibility in rural areas, with a focus on patient consent and privacy.	-“Bringing the benefits of AI healthcare LLMs to rural populations raises important questions around equitable access and accountable deployment.”
7. Ethics of LLM Education in Clinical Settings	Addresses the ethical necessity of educating clinical staff about the current applications of LLM in medical practices, including implications for medical training.	-“Incorporating LLMs into clinical education demands more scrutiny to ensure that it enhances rather than detracts from the learning experience.”
8. Ethics of User Experience in LLM Healthcare Applications	Investigates the ethical dimensions of user experience in healthcare LLM tools, focusing on the balance between user input and algorithmic output.	-“My experience so far is that AI can be a false positive machine. But I’ve only used it for LVO and PE detection. It does ok at intracranial hemorrhage.”
9. LLM Training for Mental Health: Ethical Considerations	Looks at the ethical implications of using LLM training data in supporting mental health treatments, including ADHD and other disorders.	- “Mental health LLMs require exceptionally thoughtful development and monitoring to avoid codifying outdated assumptions harmful to vulnerable groups.”
10. Ethical Aspects of LLM application in Diagnostics	Explores the ethical considerations in the LLM-driven diagnosis of health problems, focusing on accuracy, bias, and patient outcomes.	-“If applied to medical imaging diagnostics, LLMs would require extensive validation and ongoing monitoring to avoid missed or spurious diagnoses.”
11. LLM Fairness and Ethics in Healthcare	Discusses the crucial ethical issue of fairness in LLM applications within healthcare, especially in ensuring equitable treatment for all patient demographics.	-“LLMs promising to improve healthcare efficiency must not deprioritize delivering quality care equitably across patient populations.”
12. Ethical Dimensions of LLM in Public Healthcare Resources	Focuses on the ethical implications of LLM in improving the accessibility and availability of public healthcare resources.	- “Allocating LLM resources in public healthcare poses questions about prioritization and access, ensuring technology benefits the many rather than the few.”
13. Trust and Ethics in Healthcare LLM Systems	Addresses the ethical concerns related to the trustworthiness and reliability of LLM systems in healthcare settings.	- “If LLMs inserted into healthcare processes seem like black boxes, it could impede trust-building process in technology and stifle realizing potential benefits.”
14. Ethics of LLM in Enhancing clinical Workflows	Examines the ethical considerations of integrating LLM into clinical workflows, including issues related to protocol compliance and the impact on nursing practices.	“What about an AI that summarizes all the clinic visits or admissions? Or tells you about progression of disease based on several CT scans or tells you the patients hasn’t been filing meds for XYZ reasons. Throw in some risk factor calculators and you got yourself a powerful tool in diagnosis and managing your daily workflow.”

Next, we grouped these 14 themes into four higher-order ethical domains. Consolidating specific themes into the four ethical domains involved an iterative qualitative process examining the themes, identifying relationships between them, grouping conceptually related themes, and reaching full consensus amongst the three annotators on the final broader categories and allocation of themes. To develop the four ethical domains, we used the following frameworks: Principles of Biomedical Ethics framework emphasizes four central bioethical principles of autonomy, beneficence, non-maleficence, and justice. This framework can be applied to the ethical evaluation of LLM applications in healthcare, particularly concerning performance, communication, and diagnostics, ensuring that these technologies benefit patients without causing harm and respecting patient autonomy and justice [31]. Therefore, themes 1, 2, 5, 10 and 14 are grouped in domain (1) The FAIR data principles (Findability, Accessibility, Interoperability, and Reusability) are best practices for handling sensitive health data that could apply to mental care and rural contexts [32]. Themes 3, 4, 6 and 9, directly related to coding and data governance for special case such as rural healthcare applications and mental health are grouped in domain (2) The Social Determinants of Health framework promoted by the World Health Organization (WHO) underscores the importance of addressing the conditions in which people are born, grow, live, work, and age. This framework supports the domain of health equity by highlighting the need for LLM applications to promote fair access to healthcare services and to address disparities in health outcomes [33]. Themes 11 and 12 directly related to fairness and public health accessibility are grouped in doain (3) The Trustworthy AI Framework outlines seven key requirements for AI systems, including human agency and oversight, diversity, non-discrimination, and fairness. This framework supports the domain focusing on education, user experience, and trust, emphasizing the need for LLM systems to be designed and deployed in a trustworthy manner that respects human rights [34]. Themes 7, 8 and 13 directly discuss items related to user experience,, education and trust in LLM for healthcare applications, are grouped under domain (4) Table 2 shows the four core ethical domains.

**Table 2:** Core ethical domains

Core ethical domains	Themes involved
1. Ethical implications in clinical LLM applications: performance, communication, and diagnostics	-LLM-enhanced healthcare communication -LLM in nursing and care quality improvement -LLM in emergency care: ethical perspectives -Ethical aspects of LLM application in diagnostics ethics of LLM in enhancing clinical workflows
2. Ethical coding and data governance in healthcare LLM: special cases of mental care and rural healthcare applications	-Ethical monitoring of LLM coding in healthcare -Privacy ethics in LLM-enabled medical data -LLM training for mental health: ethical considerations - Ethical challenges in LLM-powered rural healthcare
3. LLM in health equity: the distribution of public health services and accessibility	- LLM fairness and ethics in healthcare - Ethical dimensions of LLM in public healthcare resources
4. Education, user experience, and trust in healthcare LLM: the relationship between users (human) and LLM systems (machine)	-Ethics of LLM education in clinical settings -Ethics of user experience in LLM healthcare applications -Trust and ethics in healthcare LLM Systems

## Discussion

### Key ethical themes identified

This study identified key ethical themes concerning the integration of LLMs in healthcare, drawing particularly from the perspectives of clinicians in online discussions. Our findings found 14 distinct themes further categorized into four higher-order ethical domains: ethical implications in clinical applications, ethical coding and data governance, health equity, and the relationship between users and LLM systems. These themes mainly cover concerns about transparent and fair LLM decisions, privacy issues, access disparities, user experiences, and the reliability of LLMs in clinical settings. These themes highlight multifaceted ethical challenges that must be addressed to ensure the responsible deployment of LLMs in healthcare.

### Ethical implications in clinical LLM applications

The identified themes and domains suggest that clinicians are generally concerned about the ethical implications of LLM integration in various aspects of healthcare, including direct patient care and communications. These concerns emphasize the need for robust ethical guidelines and frameworks that alleviate these diverse concerns to ensure LLMs’ responsible and effective use in clinical practice. Identifying specific themes provides a structured understanding of the ethical implications, guiding the development of targeted policies and practices. Moreover, the identified themes, such as LLM-enhanced healthcare communication, LLM in nursing and care quality improvement, and ethical aspects of LLM application in diagnostics, are in line with previous studies that have discussed the potential of AI to improve patient outcomes, optimize processes, and democratize healthcare access [15, 16].

What sets our study apart is the specific focus on clinicians’ voices, which are often underrepresented in discussions about AI ethics. While studies by He et al. [6] and Tian et al. [7] broadly discuss LLMs’ technical and ethical challenges, our research dives into the ethical concerns perceived by practicing clinicians. This clinician-centric approach provides practical insights into real-world implications and the ethical considerations that directly impact patient care, and complements the broader discussions in existing studies by Esmaeilzadeh [35] on the ethical challenges of AI (such as LLMs) in healthcare.

A prominent theme centered on the potential for miscommunication and misunderstanding when LLMs are used to facilitate healthcare communication. Previous studies highlight the possibility of miscommunication due to using LLMs for healthcare purposes [36, 37]. Concerns were raised regarding the ability of LLMs to navigate the touches of human language and effectively convey sensitive medical information. Similarly, the potential for overreliance on LLMs in emergency and outpatient settings was a recurring theme. Participants expressed anxieties about compromising patient safety and treatment efficacy by relying on imperfect algorithmic assessments, particularly in critical situations. Key areas of LLM application include healthcare communication through tools like chatbots and virtual assistants, which transform patient engagement by providing personalized information and support. For example, studies have shown that LLM chatbots effectively deliver health education and foster more interactive patient communication [38].

In nursing, LLM’s predictive analytics can be used to detect patients at risk and enhance care quality [39]. In emergency care, LLM can aid in swift and accurate decision-making, which is essential to prioritizing treatment and diagnosing conditions quickly, exemplifying how LLM integration can optimize care delivery and patient outcomes efficiently [40]. Moreover, clinicians found it essential that the diagnostic process involving LLMs is transparent and that the reasoning behind their recommendations is explainable. Transparency and explainability are emphasized in the literature as vital factors that can build trust and accountability in clinical decision-making [41].

### Ethical coding and data governance

The issue of bias in the coding and training data of large language models (LLMs) emerged as a significant concern among clinicians. They underscored the necessity for stringent ethical oversight and robust standards to prevent LLMs from perpetuating existing biases within healthcare systems. Aligned with prior studies, our findings also highlight the potential for privacy breaches and underscore the critical need for rigorous data security measures when handling sensitive medical data for LLM training [35].

Despite these concerns, participants acknowledged several potential benefits of LLMs in specific areas. Notably, themes emerged around the potential of LLMs to enhance the quality of mental healthcare and improve healthcare accessibility in rural regions. Clinicians also noted the capability of LLMs to optimize processes such as triage and screening. These insights provide valuable guidance for researchers and developers focusing on LLM applications in healthcare.

A key takeaway is the imperative for transparency, interpretability, and explainability in the design of AI-powered tools, including LLMs, in healthcare settings. Healthcare professionals must comprehend the rationale behind LLM outputs to ensure the technology is utilized effectively and ethically [42]. Furthermore, robust data governance practices are essential to mitigate the risks of bias and privacy violations, thereby ensuring the integrity and trustworthiness of LLM applications.

Our thematic analysis not only confirms but also expands upon concerns highlighted in recent studies, particularly regarding data privacy as a critical ethical challenge in deploying generative AI and LLMs in healthcare [43]. Echoing the findings of Chang et al. [4] and Thirunavukarasu et al. [5], our study underscores the pressing need for comprehensive ethical guidelines in the development and deployment of LLMs.

The themes related to ethical coding and data governance, especially within the contexts of mental healthcare and rural healthcare applications, resonate with existing literature on privacy, data security, and the necessity for rigorous oversight in AI development [32, 33]. Our study adds to this discourse by elucidating the ethical challenges specific to LLM applications in these sensitive healthcare domains.

In the realm of mental healthcare, clinicians expressed profound concerns about the confidentiality of patient-LLM interactions. The use of chatbots in mental health, for instance, could pose serious privacy issues, particularly regarding the retention and use of deeply personal information shared during therapy sessions. This highlights the unique challenges of implementing LLMs in mental health contexts, where patient trust and data sensitivity are of paramount importance.

Our study also revealed significant ethical dilemmas in rural healthcare applications related to data representation and model fairness. LLMs primarily trained on urban patient data may fail to accurately represent the health conditions and socioeconomic factors prevalent in rural communities. This potential bias underscores the need for diverse and representative training data to ensure equitable healthcare delivery across different geographic settings.

Furthermore, our findings emphasize the ethical implications of data governance in the telemedicine applications of LLMs. For example, when LLMs are employed to analyze video consultations or patient messages, concerns arise regarding the ethical management of patient consent and data ownership across state or national boundaries. This example illustrates the intricate interplay between LLM technology, data protection regulations, and the increasingly global nature of healthcare delivery.

### Health equity and access disparities

The themes around LLM in health equity and the distribution of public health services align with the growing recognition of the potential for AI to exacerbate or mitigate health disparities [33, 34]. Our findings underscore the importance of addressing these ethical dimensions to ensure that LLM applications promote equitable access to healthcare services. The integration of LLMs in public health has the potential to significantly impact various aspects of health equity, from improving access to information and resources in underserved communities to tailoring healthcare interventions that meet the specific needs of diverse populations. LLMs can be programmed to identify and highlight disparities in healthcare delivery, enabling public health officials and policymakers to make more informed decisions that target and reduce these inequities.

However, there is also a risk that if not carefully managed, LLMs could reinforce existing biases and disparities. For instance, if the training data for LLMs predominantly represents the experiences and needs of more privileged groups, the resulting applications may not adequately serve or may even disadvantage marginalized populations. Therefore, it is crucial to ensure that the development and deployment of LLMs in healthcare are guided by principles of fairness and inclusivity, and involve continuous monitoring and evaluation to prevent unintended consequences.

By addressing these ethical considerations, our study contributes to a deeper understanding of how LLMs can be leveraged to support health equity and improve the distribution of public health services. This includes advocating for the involvement of diverse communities in the development process, ensuring transparency in AI decision-making, and fostering collaborations between technologists, healthcare providers, and patients to create AI systems that truly serve the needs of all populations.

### Education, user experience and trust in healthcare

The themes associated with education, user experience, and trust in healthcare LLM systems resonate with the emphasis in the literature on the need for explainable, transparent, and trustworthy AI systems in healthcare [44]. Our study contributes to this discussion by highlighting the perspectives of clinicians on the ethical aspects of their relationship with LLM systems.

Several studies emphasize the importance of educating clinical staff and professionals about LLM applications in medical practices, ensuring they are aware of both these technologies’ potential and limitations [45]. Training can cover how LLM tools work, their potential benefits, potential biases, errors, and ethical considerations [46]. This education is crucial for practitioners to use LLM tools effectively, responsibly, and ethically. Our research supports the growing call for LLM literacy among healthcare professionals, highlighting the importance of continuous education for users in clinical settings to keep pace with rapidly evolving LLM technologies [47].

Participants reported ambivalence about AI-assisted decision-making, reflecting concerns about maintaining clinical autonomy and the potential for deskilling. This links to broader discussions in the literature about preserving the art of clinical reasoning in an increasingly technology-driven healthcare environment [48]. Thus, physicians must ensure that reliance on LLMs and AI does not erode our ability to think critically and independently.

The introduction of LLM can augment, not replace, the essential elements of healthcare delivery [49]. This entails carefully integrating LLM tools to support healthcare professionals, allowing them to focus more on the interpersonal aspects of patient care. AI’s role, primarily in data analysis, diagnostics, and treatment recommendations, should complement the healthcare provider’s expertise [50]. Maintaining the human touch in patient-provider interactions is crucial, as is preserving empathy and understanding, which are fundamental to patient care. As LLM systems become more integrated into healthcare settings, they should be designed and implemented to bolster these human elements rather than overshadow them. LLMs could transform the way healthcare providers communicate with patients. It is necessary to ensure that this technology supports and enhances this relationship rather than undermining it with impersonal or inaccurate communication. Lastly, integrating LLM into clinical workflows is a significant shift in care delivery. By automating routine tasks, LLM may allow clinicians to focus more on direct patient care, enhancing the overall quality of healthcare services. This is evident in applications like LLM-driven medication management, which not only reduces the risk of human error but also improves efficiency in patient care [51].

### Theoretical contributions

Theoretically, our study contributes a structured, empirically grounded framework for understanding key ethical implications of LLM integration in healthcare from the perspective of online clinician communities. The identified themes and domains provide a structured foundation for further research and theory development in this emerging field. Also, by focusing on the voices of clinicians, our research offers a unique theoretical lens that emphasizes the practical ethical concerns faced by healthcare providers. This perspective is crucial for developing ethically sound AI systems sensitive to the realities of clinical practice.

### Practical implications

Practically, our findings can inform the development of ethical guidelines, standards, and policies for the responsible integration of LLMs in healthcare. The insights from clinicians can guide the design and deployment of LLM systems that are not only technically robust but also ethically aligned with the values and concerns of healthcare professionals and patients. Policymakers can use these insights to create regulations that ensure LLMs are used responsibly, minimizing risks to patients. Developers of LLMs can also use the insights from this study to design systems that are more transparent, fair, and aligned with the ethical expectations of clinicians. This alignment can enhance the trust and acceptance of AI tools in healthcare settings. Moreover, Our findings highlight the need for comprehensive training programs for clinicians on the ethical use of LLMs. By understanding the potential ethical pitfalls, clinicians can better navigate the complexities of integrating these technologies into patient care.

### Limitation and future study

While our study provides valuable insights, it also has limitations that suggest avenues for future research. This study, focusing on the ethical aspects of LLM in healthcare, reveals several avenues for future research due to its limitations. We utilized a dataset exclusively sourced from a subreddit frequented by self-identified physicians and healthcare professionals, who signal their roles within the healthcare ecosystem through specific flair tags next to their usernames. This approach, while innovative, suggests the potential for more robust data collection in future research by directly engaging with clinicians. Expanding the scope to include diverse forums could offer a richer, more varied perspective of clinician viewpoints from different regions and medical specialties. Future investigations should also consider a comparative analysis across various clinician communities to deepen the understanding of these issues.

Moreover, future studies may expand the scope to include the perspectives of patients, technology developers, policymakers, and ethicists, providing a more holistic view. Incorporating perspectives from patients, tech developers, policymakers, and ethicists will give a more holistic view of the ethical landscape. Further research could explore potential solutions in regulation and policy, extending beyond identifying ethical implications. We also acknowledge that the keywords used for data extraction may not have been exhaustive. Future research should aim to address these gaps by incorporating a more extensive set of keywords to capture a broader range of discussions and ethical considerations pertaining to a variety of LLMs tailored to medical contexts .

In addition, while our team reached a consensus on qualitative coding, engaging a broader range of experts could diversify thematic insights, emphasizing the subjectivity in qualitative research and the potential for biases inherent in human interpretation. While topic modeling is well-supported in literature for applications such as corpus exploration and information retrieval, it is crucial to prioritize evaluations based on real-world task performance over merely optimizing traditional likelihood-based measures. To bridge the gap between automated evaluations and human interpretability, future developments in topic modeling should consider incorporating human judgments directly into the model learning process. Alternatively, developing computational methods that simulate human evaluations could further enhance the relevance and usability of the topics generated, making them both qualitatively rich and practically useful. Lastly, given the rapid advancements in LLM technology and its healthcare applications, ongoing reassessment of the ethical landscape is essential. Longitudinal studies are recommended to observe evolving clinician perspectives as technology integration progresses, ensuring that ethical considerations remain aligned with technological developments.

## Conclusion

This study develops a framework from self-identified clinician insights to categorize the ethical challenges of integrating LLM in healthcare, identifying 14 key themes. These themes cover issues spanning transparent and fair LLM decisions, privacy, access disparities, user experiences, and reliability concerns that must be proactively addressed to harness LLM’s immense potential while respecting patient rights. As LLM capabilities rapidly progress, sustained ethical inquiries focusing on real-world integration complexities from stakeholders’ viewpoints remain imperative to responsible innovation. Our thematic mapping notably synthesizes, reinforces, and expands current discourse at the intersection of medicine and LLM domain, advocating for tailored governance rather than broad regulations. This research enriches the ethical groundwork to guide policy and practice, promoting the use of LLM in healthcare to improve clinical outcomes ethically and effectively.

## Electronic supplementary material

Below is the link to the electronic supplementary material.

Supplementary Material 1

## Data Availability

The data underlying this article will be shared on reasonable request to the corresponding author.
