# Supplementary material for: Clinician voices on ethics of LLM integration in healthcare: a thematic analysis of ethical concerns and implications
Source: BMC Med Inform Decis Mak. 2024 Sep 9;24:250. doi: 10.1186/s12911-024-02656-3 (PMC11382443; doi:10.1186/s12911-024-02656-3)
Supplement: Supplementary file 1 — Supplementary Material 1 [file 12911_2024_2656_MOESM1_ESM.docx]

# Supplemental material

List of keywords

“AI”, “OpenAI”, “artificial intelligence”, “ChatGPT”, “Chat GPT”, “GPT3”, “GDT-3”, “GPT 4”, “GPT-4”, “LLM”, “Large Language Models”, “Natural Language Processing”, “AI language models”, “conversational AI”, “text generation”, “LLM in healthcare”, “AI writing tools”, “privacy”, “transparency”, “bias”, “machine learning fairness”, “accountability”, “safety”, “security” , “social impact”, “algorithmic bias”, “regulation”, “moral implication”, “ethical”, “ethics”, “ethical AI.”

Note about data collection and processing

Using predefined keywords associated with large language models (LLM) and artificial intelligence (AI), we initially extracted 10,374 posts and comments. During this extraction process, we encountered duplicate comments attributable to Reddit's comment structure. Upon removing these duplicates, the dataset was reduced to 6,459 comments. Additionally, we identified and excluded comments flagged as deleted or removed in accordance with the regulations of the subreddit "medicine," further reducing the dataset to 6,141 comments.

Subsequently, we concentrated on comments specifically addressing the ethics of LLM and AI, employing relevant keywords to ensure alignment with our research question. Following this final filtration, we obtained a total of 3,049 posts and comments pertinent to our research objectives. To prepare a clean corpus suitable for topic modeling, we performed stopword removal and lemmatization. This data cleaning process was meticulously designed to retain all meaningful words without eliminating any crucial information. The removal of stopwords and the application of lemmatization are standard practices in natural language processing, ensuring that the corpus is optimized for subsequent analysis.

We utilized Latent Dirichlet Allocation (LDA) to identify latent topics within the dataset. These topics represent clusters of words that frequently co-occur, thereby indicating common themes in the data. The term “topic” refers to these clusters. We then reviewed these topics to ensure they accurately reflected the underlying data and assigned thematic labels based on the content and context of the posts.

Table A. Expanded definition of themes and ethical implications and concerns:

| **Themes** | **Topics** | **Definition and ethical implications** | **Potential ethical concerns:** |
| --- | --- | --- | --- |
| LLM-Enhanced Healthcare Communication | -LLM facilitates communications about home visits and insurance  -LLM facilitates patient-provider Interactions  -LLM facilitates communications about Narcan and relapse cases | Explores ethical concerns surrounding using LLM in facilitating communication for healthcare purposes, including patient-provider interactions and discussions of preventive measures. While patient-provider communication is a central focus, the communication can cover any healthcare stakeholder interactions facilitated by LLMs, such as patient-caregiver, doctor-doctor, and patient-insurer. | Factual inaccuracy: A key concern would be that LLMs could provide patients with incorrect or misleading medical advice if they are not properly trained, validated, and approved for each use case. This could clearly put patients at risk for harm.  - Impacts on empathy: LLMs may fail to convey appropriate empathy, compassion, and emotional intelligence compared to human clinicians during sensitive conversations. Some also worry LLMs may manipulate emotions. Both could erode trust or healing.  - Depersonalization of care: Using LLMs in patient-provider communication could lead to a depersonalized healthcare experience. Patients might feel less valued or understood if their interactions are primarily with an AI system rather than a human healthcare provider.  - Informed consent: Using chatbot LLMs for health purposes without proper identity disclosure and consent raises ethical issues regarding transparency and autonomy.  - Healthcare disparities: Relying on LLMs for patient communications risks disproportionately benefiting more privileged populations if the tools are not suitably validated across user demographics. |
| LLM in Nursing and Care Quality Improvement | -LLM can improve nurses’ job performance.  -AI recommendation agent can improve the quality of care, reduce the burden of decision making for care providers  -LLM provides support for primary care  -LLM with a little control can be helpful in clinical practices and saving time | Focuses on the ethical aspects of LLM's role in nursing, such as improving job performance and care quality, while considering the reduced decision-making burden on care providers.  Potential benefits:  - Improving nurse job performance and reducing burnout by automating certain documentation/administrative tasks. This could allow nurses to spend more time delivering high-quality patient care.  - Enhancing care quality and patient health outcomes by leveraging LLM capabilities in areas like symptom checking, treatment recommendations, etc. | - Overreliance on LLM recommendations could erode nurses' knowledge/judgment over time. This could negatively impact care quality if nurses become less skilled clinically.  - Accountability questions if LLM recommendations related to patient care are inaccurate or harmful. Who bears responsibility - the LLM developer, the health system implementing it, or the individual nurse?  - Nurse displacement or impacts on the workforce due to automation of certain tasks previously performed by nurses. Could exacerbate existing nursing shortages. |
| Ethical Monitoring of LLM Coding in Healthcare |  | Discusses the need for ethical guidelines and standards in developing and coding LLM tools in healthcare. The term coding refers to the process of designing, programming, and "coding" the software components that enable LLMs and AI-based tools to perform various prediction, treatment recommendation engines, AI chatbots, algorithmic diagnosis systems, and documentation tasks related to healthcare. | - Algorithmic bias: There is concern that deficiencies or biases in the training data or development process for healthcare LLMs could result in biased performance, such as higher error rates or worse recommendations for underrepresented patient populations. This could exacerbate healthcare disparities. Creating standards to test for and address algorithmic bias is important.  - Transparency: LLMs and AI systems often lack transparency in how recommendations/predictions are generated, functioning more as "black boxes." This lack of model interpretability makes evaluating their reasoning and safety difficult. Ethical guidelines should emphasize transparent processes.  - Privacy protection: Accessing the large datasets needed to train healthcare LLMs raises privacy risks if personal health data is not adequately protected, anonymized, or consented to for research use. Data security and protection standards are imperative.  - Goal misalignment: With healthcare LLMs often developed by private or governmental entities beyond direct provider control, their embedded goals, incentives, and optimization targets may not fully align with promoting holistic patient well-being and ethical care delivery as the top priorities. Guardrails could help address this issue. |
| Privacy Ethics in LLM-Enabled Medical Data |  | Highlights the ethical concerns related to privacy and data security in the context of LLM accessing sensitive medical lab results. | - Breaches and unauthorized access: Perhaps the most evident concern is the risk of healthcare LLMs or the datasets used to train them being breached or hacked, enabling unauthorized third parties to access confidential patient information. Preventing such breaches is an obvious priority, yet challenges persist in keeping pace with threats.  - Lack of informed consent: Patients may be unaware of when and how their medical lab results, doctor's notes, or other health data get incorporated into health system datasets used to develop healthcare-focused LLMs. Their consent and transparency around data usage practices matter ethically.  - Securing highly sensitive data: Granular health data found in areas like mental health evaluations, sexual health conversations, and lab test outcomes requires especially heightened security and governance standards given potential individual or societal harms if improperly accessed or shared.  - Commercial exploitation of data: Profit-motivated partnerships between healthcare systems and private LLM developers carry risks of patient data being utilized primarily to benefit business objectives rather than the patients themselves, eroding trust in data stewardship. |
| LLM in Emergency Care: Ethical Perspectives |  | Examines the ethical considerations in using LLM for emergency and outpatient treatment, emphasizing patient safety and treatment efficacy. | - Patient safety risks: The top concern is that LLMs make inaccurate diagnoses or make treatment recommendations that harm patients in high-stakes emergency/outpatient contexts where risks of harm are greater without proper physician oversight.  - Over-reliance leading to skill erosion: Doctors becoming overly dependent on LLM treatment suggestions could erode their critical thinking skills over time, negatively impacting care quality if LLMs underperform or errors occur.  - Liability & legal accountability: If an LLM makes a negligent diagnosis/treatment recommendation that injures a patient, major questions emerge on legal responsibility. Is the clinician still liable, or is the LLM developer/deployer?  - Exacerbating healthcare disparities: Insufficient regulatory safeguards could enable emergency care algorithms to differentially underperform for marginalized patient groups if not rigorously validated, raising discrimination issues.  - Informed consent & trust erosion: Patients may object to unknown AI systems influencing emergency treatment decisions without permission if care teams over-rely on LLMs without sufficient identity disclosure and consent processes. |
| Ethical Challenges in LLM-Powered Rural Healthcare |  | Explores ethical questions surrounding using LLM to enhance healthcare accessibility in rural areas, with a focus on patient consent and privacy. | - Patient privacy: Rural patients may have heightened privacy sensitivities about personal health details if LLMs enable wider data sharing with urban centers. Obtaining meaningful consent and encrypting/anonymizing data is vital.  - Equal access to benefits: Any LLM integration must prioritize expanding access and quality of care for rural areas themselves rather than merely benefiting urban hubs' training algorithms on rural data. Equity is key.  - Transparency: Clearly communicating to rural patients when an LLM may be involved in critical care decisions rather than a physician directly is important for trust and informed consent.  - Connectivity requirements: Reliance on internet-based LLMs could exclude the most disconnected rural residents struggling with poor broadband access. Alternatives respecting this divide warrant consideration.  - Community governance: Rural communities should have representation in healthcare AI ethics boards and standard-setting bodies helping govern LLM integration in a way that aligns with rural health priorities. |
| Ethics of LLM Education in Clinical Settings | -Clinical staff need to be educated about Up-to-date application of AI in medical practices  -LLM role in medical student education journey | Addresses the ethical necessity of educating clinical staff about the current applications of LLM in medical practices, including implications for medical training. | - Informed consent: Clinicians have a right to understand where, when, and how LLMs are utilized in the care delivery process. Lack of transparency around LLM usage could undermine informed consent from both clinicians and patients.  -Clinical skill erosion: Overreliance on LLMs for diagnostic and treatment recommendations risks eroding core clinical skills, knowledge, and critical thinking abilities over time if clinicians become overdependent on algorithmic insights versus building experience themselves.  - Liability ambiguity: Without proper education on appropriate vs inappropriate uses of healthcare LLMs along with limits of these tools, clinicians may unintentionally misuse or become legally vulnerable from problematic overreliance if harm results.  - Exacerbating disparities: Lack of clinician education around risks of algorithmic bias, the need to scrutinize LLM performance differences across patient demographics, and when human judgment is superior could perpetuate discrimination.  - Patient trust: Patient trust in their providers may deteriorate if clinicians appear over-reliant on opaque LLMs for sensitive health decisions rather than conveying sound clinical judgment patients can rely on. |
| Ethics of User Experience in LLM Healthcare Applications |  | Investigates the ethical dimensions of user experience in healthcare LLM tools, focusing on the balance between user input and algorithmic output. | - Overreliance on automation: Prioritizing seamless user experience by having an LLM generate diagnoses/recommendations with minimal user input risks clinician overreliance and skill erosion over time if they become passive recipients rather than actively engaged.  - Lack of transparency and explainability: Designing LLMs that function more like inscrutable "black boxes" with limited transparency around how recommendations are generated infringes on user autonomy and informed consent in applying outputs.  - Perpetuating biases: Over-automation without sufficient clinician input and oversight to catch potential algorithmic biases that may emerge in patient assessments and treatment decisions could propagate discrimination.  - Disconnecting care teams: If LLMs fully automate communication of insights to patients without shared discussion of recommendations among their interdisciplinary care team, this could disrupt care coordination and patient understanding.  - Loss of human empathy: Patients could feel less heard or emotionally supported if their complex health narratives rely strictly on emotionless LLM apps versus compassionate human clinicians actively listening. |
| LLM Training for Mental Health: Ethical Considerations |  | Looks at the ethical implications of using LLM training data in supporting mental health treatments, including ADHD and other disorders. | - Patient Privacy: Mental health conversations contain highly sensitive personal information. Adequately deidentifying, securing, and getting consent for psychotherapy dialogues used to train LLMs is critical to respect privacy.  - Representation Biases: LLMs trained on limited demographic data risk perpetuating biases against marginalized groups if the algorithms underperform in supporting diagnosis/treatment for underrepresented populations. Proactive mitigation is required.  - Clinical Relationship Impacts: Over-relying on LLMs versus direct clinician-patient dialogues could erode therapeutic alliances and the empathy critical for mental healthcare. Safeguards on use warrant consideration to prevent this.  - Informed Consent: Clearly disclosing when mental health LLMs are utilized versus a clinician alone is key for patient agency and awareness regarding vulnerabilities like privacy. Consent processes are vital.  - Clinical Skill Atrophy: Extensive utilization of diagnostic/treatment LLMs could contribute to clinician skill erosion over time if over-relied upon. Ensuring continual skills development is an important ethical priority. |
| Ethical Aspects of LLM Application in Diagnostics |  | Explores the ethical considerations in the LLM-driven diagnosis of health problems, focusing on accuracy, bias, and patient outcomes. | - Diagnostic accuracy and safety: The top priority is ensuring clinical-grade testing and oversight to confirm LLM diagnostic performance is at least as accurate as human clinicians to prevent potential patient harm from incorrect diagnoses.  - Algorithmic bias: If LLM diagnostic tools exhibit higher error rates or make consistent misdiagnoses for certain patient demographics like underrepresented minorities, it risks worsening health disparities and raises discrimination issues. Proactive bias testing and mitigation is critical.  - Overreliance: Physicians and nurses who may become over-reliant on LLMs for initial diagnoses could get deskilled over time if they are not still actively verifying each diagnostic output before treatment. Preventing clinical skill erosion warrants caution around appropriate use cases.  -Transparency: Disclosing the use of an LLM to patients in the diagnostic process and the evidence around its capabilities relative to a clinician-led diagnosis is key for informed consent purposes. Transparency builds better trust.  -Data access equality: There are open questions around whether the deployment of proprietary LLM diagnostic tools trained on extensive patient datasets may unwittingly disadvantage patient populations at healthcare systems with fewer resources. Preventing additional disparities warrants consideration. |
| LLM Fairness and Ethics in Healthcare |  | Discusses the crucial ethical issue of fairness in LLM applications within healthcare, especially in ensuring equitable treatment for all patient demographics. | -Algorithmic bias: A major priority is testing LLMs for biased performance, such as higher error rates in assessing symptoms or recommending treatments for minority groups. Without proactive bias testing, LLMs could propagate racism, sexism, ageism or other discrimination.  - Exacerbating disparities: Biased LLMs risk worsening existing health disparities if they differently underserve vulnerable communities. Regulatory assessments of bias with public reporting could incentivize guardrails before deployment.  - Unequal access: There are risks that the high cost of commercial LLMs could allow wealthy healthcare systems faster access, disadvantaging patients at low-resourced providers. Policy expansions enabling equal access warrant discussion.  - Population representativeness: LLMs trained only on subsets of the population may underperform on diverse patient needs. Requiring representative training data with testing on excluded groups could mitigate this.  - Community governance: Marginalized patient populations should have direct participation in healthcare AI ethics boards overseeing LLM integration to ensure solutions centering fairness emerge |
| Ethical Dimensions of LLM in Public Healthcare Resources |  | Focuses on the ethical implications of LLM in improving the accessibility and availability of public healthcare resources. | -Equal access to benefits: There should be an assurance that any improvements to public health resources enabled by LLMs directly benefit and are accessible to the patient populations, contributing data to develop such tools. Expanding inequality gaps would be unethical.  -Informed consent: Patients interacting with public-facing LLM-powered chatbots, symptom checkers, appointment schedulers etc., should be clearly notified an AI is involved rather than a human. Transparency enables informed consent.  -Patient privacy: Public health datasets used to develop population health LLMs must be effectively anonymized with consent and securely managed to maintain patient trust in data practices.  -Community governance: Representatives of patient communities affected by the integration of LLMs into public health resources should have seats at oversight bodies governing development to ensure patient-centeredness.  -Displacement risks: Automating certain administrative tasks with LLMs warrants analysis regarding potential healthcare job displacement effects and transition support if workforce disruption occurs. |
| Trust and Ethics in Healthcare LLM Systems |  | Addresses the ethical concerns related to the trustworthiness and reliability of LLM systems in healthcare settings. | - Clinical accuracy: Demonstrating through rigorous testing across diverse patient cohorts that recommendations meet or exceed clinician-level safety and efficacy is imperative for trusting LLM outputs for diagnosis, treatment planning, etc. Failing to establish clinical equivalence risks patient harm if LLMs underperform human providers.  - Transparency: Lack of transparency around an LLM’s capabilities, limitations, training process, and validation testing erodes trust by making the systems seem like inscrutable “black boxes”. Being transparent on aspects like accuracy rates and intended use cases is important.  - Explainability: Closely tied to transparency, LLMs that cannot explain the reasoning behind a diagnosis or treatment decision inhibit trust by clinicians and patients alike compared to human judgment. The inability to explain failures also hampers resolving issues.  - Security: Robust cybersecurity protections around patient data and vulnerability testing of LLM systems mitigate the risk of data breaches, which could dramatically deteriorate public trust. Preventing unauthorized access is key for healthcare AI.  - Liability: Unclear legal and ethical accountability if LLMs err and harm patients corrodes provider trust in deploying the tools. Resolving medical liability and clarifying human vs system responsibilities is pivotal. |
| Ethics of LLM in Enhancing Clinical Workflows |  | Examines the ethical considerations of integrating LLM into clinical workflows, including issues related to protocol compliance and the impact on nursing practices. | -Overreliance: Nurses becoming overdependent on LLM diagnostic or treatment recommendations could contribute to deskilling over time and clinical knowledge erosion if less learning occurs organically. Maintaining competencies is vital.  -Devaluation of nursing judgment: Prioritizing LLM outputs without sufficient regard for nursing insight based on holistic patient familiarity risks deteriorating the role of compassionate, relationship-based care. Nursing judgment retains integral value.  -Inflexible protocols: Strictly protocolized LLM guidance could constrain nursing autonomy and human-centered decision-making that allows for flexibility based on nuances of patient needs and circumstances. Room for situational discretion matters.  -Displacement effects: Utilizing LLMs to automate aspects of documentation, coordination, and administrative nursing workflows risks eventually replacing certain nursing roles. Assessing workforce impacts is an ethical necessity.  -Nurse well-being: While LLMs could augment some tedious duties, the effects on nurse fatigue, burnout, and overall well-being warrant evaluation to prevent unintended detriment. Optimization should consider holistic human thriving. |

Table B. Example of Quotes Collected from Subreddit Medicine.

| **Theme** | **Quotes** |
| --- | --- |
| LLM-Enhanced Healthcare Communication | -"The use of AI [LLMs] to enhance doctor-patient communication could improve outcomes, but we must consider how to prevent harms due to potential miscommunication or misunderstanding."  -"The integration of AI into healthcare communication streamlines patient-provider interactions, but it necessitates rigorous oversight to ensure accuracy and empathy are not compromised."  -"There is promise in using AI to make complex healthcare information more accessible for patients, as long as they are carefully designed to avoid oversimplification.” |
| LLM in Nursing and Care Quality Improvement | -"I had a nursing Educator during my RN residency program describe some technology similar to this [LLMs] and that it would eventually be able to chart my shift assessment for me."  -"I am hopeful about the prospects for AI to assist in care coordination, freeing up nurses for more direct patient care."  -"The use of AI for care quality improvement introduces an ethical obligation to continually validate the accuracy of the information provided to nursing staff." |
| Ethical Monitoring of LLM Coding in Healthcare | -"For LLM coding in healthcare, robust oversight must warrant algorithms are free from biases and respect patient diversity… I fear AI optimized for efficiency could worsen issues if deployed in medical coding without a framework ensuring decisions are fair."  -"I feel like AI could be used for coding queries though. Most of those are either completely asinine or telling us we forgot to document something. I feel like a computer could probably do that someday soon."  -"[AI] Coder here. Computer-Assisted Coding is not going to take my job away anytime soon. CAC is good at seeing a word and assigning a code. What it cannot do, is read." |
| Privacy Ethics in LLM-Enabled Medical Data | -"I see so many issues with confidentiality and accuracy but a lot of potential too."  -"Effective anonymization measures are essential for using private health data to train diagnosis LLMs that protect patient confidentiality."  -"While [LLMs] can enhance the analysis of medical data, moral considerations around patient consent and data anonymization are important." |
| LLM in Emergency Care: Ethical Perspectives | - "Using [AI] technology in emergency care must be approached with caution, ensuring that speed does not compromise patient safety or decent standards."  -"The time pressures of emergency response may impede full ethical vetting of new [LLM] tools, a concern requiring proactive mitigation."  -"These [LLM] tools might aid emergency triage, but safeguards are needed to prevent over-reliance on imperfect algorithmic assessments when immediate care is critical." |
| Ethical Challenges in LLM-Powered Rural Healthcare | -"Totally agreed, but I do like the idea of a [AI] machine out in rural undeserved areas at least finding folks who have retinopathy to get things started. Just another niche use case."  -"Bringing the benefits of AI healthcare LLMs to rural populations raises important questions around equitable access and accountable deployment."  -"The deployment of AI in rural settings highlights ethical concerns of technology reliance in areas with limited healthcare infrastructure." |
| Ethics of LLM Education in Clinical Settings | -“ Training a generative AI on millions and millions of patient charts in Epic plus every article and algorithm and factoid inside UpToDate then putting that into every computer terminal inside every hospital and clinic”  -"I am using it [LLM] as dictation software. HIPAA etc makes it all a little too complicated and I do not want to be the one training our patients to be comfortable with it. What I do is go back to my room and dictate a rough soap note without having to be precise about what goes in which section and the AI scribe tool organizes it. I do have to review it but way easier because I don't have to think about sentence formatting."  -"Incorporating LLMs into clinical education demands more scrutiny to ensure that it enhances rather than detracts from the learning experience." |
| Ethics of User Experience in LLM Healthcare Applications | - “It was the resident on a very busy stroke service. I chalk it up to either being new or overwhelmed. Still, the experience has stuck with me. My own radiology residents are putting too much faith in these “AI” programs. Most of them don’t even know how to read the perfusion portion anymore.”  -"My experience so far is that AI can be a false positive machine. But I’ve only used it for LVO and PE detection. It does ok at intracranial hemorrhage."  -"The effective design of LLM healthcare applications requires a patient-centered approach that prioritizes accessibility and usability." |
| LLM Training for Mental Health: Ethical Considerations | -"Utilizing generative AI and LLMs for mental health training must be ethically managed to respect the sensitive nature of psychological well-being and mental disorders."  - "Mental health LLMs require exceptionally thoughtful development and monitoring to avoid codifying outdated assumptions harmful to vulnerable groups."  -"The incorporation of AI-based applications using conversations into mental health services requires careful consideration of the impact on patient-clinician relationships." |
| Ethical Aspects of LLM Application in Diagnostics | - "I personally don't like the term 'hallucination' in this diagnostic context because I find it somewhat misleading. But it is the term used. Anyway, it is usually used in the context of LLM when it confidently provides an answer or states something that is grossly incorrect or untrue. This is a common problem with LLM for some tasks. It often surfaces when you ask it to provide exact quotes or strings of data based on its existing fund of knowledge."  -“When AI starts to integrate into diagnostics (radiology especially) would it possibly cause a big strain on resources after the success of having more patients diagnosed and needing treatment than ever before?”  -"If applied to medical imaging diagnostics, LLMs would require extensive validation and ongoing monitoring to avoid missed or spurious diagnoses." |
| LLM Fairness and Ethics in Healthcare | -"LLMs promising to improve healthcare efficiency must not deprioritize delivering quality care equitably across patient populations."  -"Ensuring fairness in LLM applications in healthcare is an ethical imperative, requiring vigilance against biases that could harm patient outcomes."  -"In developing healthcare LLMs, we have an important duty to assess the entire pipeline - from data to deployment - for sources of unfair bias against at-risk groups." |
| Ethical Dimensions of LLM in Public Healthcare Resources | -"The public is already starting to push back against non-physician [AI] providers. They want doctors and real people. AI will absolutely be incorporated into medicine and pretty much everything. It will revolutionize many things, including public health and medicine, it’s just not going to replace physicians. It’s too extreme of a take I think."  - "As with any automation employed in doling out healthcare resources, LLMs require thoughtful design and ongoing ethical checks and balances against bias."  - "Allocating LLM resources in public healthcare poses questions about prioritization and access, ensuring technology benefits the many rather than the few." |
| Trust and Ethics in Healthcare LLM Systems | - “One tool that has caught my attention is Scholar.ai. It appears to be a promising solution for accessing current and up-to-date medical literature. I reached out to a doctor who had expressed enthusiasm for Scholar.ai, and they shared some intriguing insights. However, they also mentioned a significant concern: Scholar.ai seemed to generate "hallucinations" that made it difficult to fully trust the information it provided.”  -"Building trust in healthcare LLM systems needs transparency about their capabilities and limitations to foster informed patient consent."  - “If LLMs inserted into healthcare processes seem like black boxes, it could impede trust-building process in technology and stifle realizing potential benefits." |
| Ethics of LLM in Enhancing Clinical Workflows | - What about an AI that summarizes all the clinic visits or admissions? Or tells you about progression of disease based on several CT scans or tells you the patients hasn’t been filing meds for XYZ reasons. Throw in some risk factor calculators and you got yourself a powerful tool in diagnosis and managing your daily workflow.  -"Nursing insight must inform LLM implementation affecting healthcare roles, not top-down automation blind to realities facing nurses and impacts to patient relationships."  -"Designing AI-enabled tools to improve nurse workflows requires deliberate analysis to avoid overloading or devaluing those delivering essential patient care." |

# Further Discussions:

***Using AI by hospitals seeking to improve their bottom line***

We should acknowledge that none of the themes explicitly focus on using AI for profit. Although it is not directly explicit to our themes, we can discuss that several of the extracted themes will lead to profit motives. For instance, hospitals may utilize LLMs to streamline nursing workflows and reduce staffing costs, even if they are not primarily pursuing care quality gains. Partnerships with private LLM developers could lead to patient data use benefiting business objectives over patients. Also, implementing diagnostic/triaging LLMs could connect to incentives around patient throughput and revenues. Thus, in the last section, we describe AI implementation in healthcare from profit and cost perspectives.

**The profit motive of AI companies**: Companies that develop AI and ML technologies, such as OpenAI, have financial goals. When these technologies are applied in healthcare, there is a concern that the primary motive could shift from patient welfare to profit generation. Ethically, this raises questions about whether the development and deployment of such technologies are driven by improving patient outcomes or by the potential for financial gain.

**Healthcare organizations seeking profit**: Hospitals and healthcare providers might use AI to improve efficiency and reduce costs. While this can be beneficial, there is a risk that the primary focus could become profit maximization rather than patient care. This could lead to decisions that prioritize financial considerations over patient needs, such as understaffing or reducing the duration of patient stays without considering individual patient needs.

**Direct-to-consumer advertising**: AI can be used to target consumers with advertisements for healthcare procedures. This raises ethical concerns about manipulating patient choices and promoting unnecessary treatments. Using sophisticated algorithms to analyze patient data and predict which patients might be receptive to certain procedures could increase elective procedures that are more profitable but not necessarily in the patient's best interest.

**Upcoding and billing practices**: AI might be used to optimize billing practices, including upcoding, where providers bill for more expensive services than were actually provided. This not only has ethical implications but also legal ones, as it constitutes healthcare fraud. Using AI in this manner would be a clear example of putting profit over patient welfare.

**Access and inequality**: The high cost of developing and implementing AI solutions in healthcare can increase healthcare costs, potentially limiting access to these advanced technologies for underfunded hospitals or less affluent patients. This could exacerbate existing inequalities in healthcare access and outcomes.

**Resource allocation**: AI could be used to allocate limited healthcare resources. While this can improve efficiency, there is a risk that these decisions could be driven by cost-effectiveness rather than patient need, potentially disadvantaging certain groups of patients
